# Functional Characterization of a Novel Oxidative Stress Protection Protein in the Pathogenic Yeast *Candida glabrata*

**DOI:** 10.3389/fgene.2020.530915

**Published:** 2020-09-25

**Authors:** Jane Usher, Yogesh Chaudhari, Victoria Attah, Hsueh-lui Ho, Ken Haynes

**Affiliations:** ^1^Medical Research Council Centre for Medical Mycology, University of Exeter, Exeter, United Kingdom; ^2^School of Biosciences, University of Exeter, Exeter, United Kingdom; ^3^Hawkesbury Institute for the Environment, Western Sydney University, Penrith, NSW, Australia

**Keywords:** *Candida glabrata*, oxidative stress, SGA, human fungal pathogens, comet assay (Single cell gel electrophoresis)

## Abstract

Candida species are important pathogens of humans and the fourth most commonly isolated pathogen from nosocomial blood stream infections. Although Candida albicans is the principle causative agent of invasive candidosis, the incidence of *Candida glabrata* infections has rapidly grown. The reason for this increase is not fully understood, but it is clear that the species has a higher innate tolerance to commonly administered azole antifungals, in addition to being highly tolerant to stresses especially oxidative stress. Taking the approach that using the model organism, *Saccharomyces cerevisiae*, with its intrinsic sensitivity to oxidative stress, we hypothesized that by expressing mediators of stress resistance from C. glabrata in *S. cerevisiae*, it would result in induced resistance. To test this we transformed, en-masse, the *C. glabrata* ORFeome library into *S. cerevisiae*. This resulted in 1,500 stress resistant colonies and the recovered plasmids of 118 ORFs. Sequencing of these plasmids revealed a total of 16 different C. glabrata ORFs. The recovery of genes encoding known stress protectant proteins such as GPD1, GPD2 and TRX3 was predicted and validated the integrity of the screen. Through this screen we identified a C. glabrata unique ORF that confers oxidative stress resistance. We set to characterise this gene herein, examining expression in oxidative stress sensitive strains, comet assays to measure DNA damage and synthetic genetic array analysis to identify genetic interaction maps in the presence and absence of oxidative stress.

## Introduction

Biological systems function in constantly changing complex environments, where they are subject to wide ranging perturbations. Their ability to adapt to these perturbations is essential for all life and understanding the mechanisms that underpin these adaptations are of fundamental biological interest. Although, *Candida albicans* is the principle causative agent of invasive candidosis, the incidence of *Candida glabrata* infection has grown rapidly over the last 20 years, and it is now responsible for approximately 25% of cases (Hajjeh et al., 2004). The reason for this increasing incidence of *C. glabrata* infection is not fully understood. The molecular mechanisms underpinning the response to stress is relatively well characterized in a number of model and pathogenic species (Gellon et al., 2001; Lackner et al., 2012; Boiteux and Jinks-Robertson, 2013; Miramón et al., 2013; Storr et al., 2013), but remains relatively unexplored in *C. glabrata*.

When engulfed by phagocytic cells pathogenic fungi are submitted to combinations of stress that cause oxidative damage, which if not repaired can result in cell death (Fang, 2004). *C. glabrata* is highly resistant to oxidative stress conditions, having evolved capabilities to cope with such stress (Nikolaou et al., 2009; Roetzer et al., 2011). *C. glabrata* can persist for long periods as a human commensal, and live cells can be isolated weeks after infection (Brieland et al., 2001; Jacobsen et al., 2010), clearly demonstrating that *C. glabrata* can adapt to the stresses encountered within the host. Indeed, *C. glabrata* has been shown to survive and multiply inside murine phagocytic cells (Kaur et al., 2007; Roetzer et al., 2008; Seider et al., 2011). When engulfed by phagocytes, *C. glabrata* cells are exposed to reactive oxygen species (ROS) plus cationic fluxes activated by intracellular ion currents (Fang, 2004). The mechanistic basis of *C. glabrata* stress resistance is not understood but genes encoding functions in cell wall biosynthesis, nutrient acquisition, metal (calcium and iron) ion homeostasis and stress response have been implicated by functional genomics (Seider et al., 2014). Previous studies have demonstrated that the *C. glabrata* response to *in vitro* exerted oxidative stress is very similar to that observed upon phagocyte engulfment, both at the level of gene expression (Kaur et al., 2007; Fukuda et al., 2013), where the up-regulation of genes encoding functions related to stress adaptation and nutrient recycling overlap substantially, and in growth kinetics (Kaloriti et al., 2012) where in both environments, approximately 20% of *C. glabrata* cells survive initial contact with a substantial delay occurring prior to growth re-commencing. These data sets demonstrate that *in vitro* oxidative stress is a realistic model of host-induced stress and the ability to survive oxidative stress is an important virulence determinant for pathogens. Understanding this network, and the role that selected components play in stress resistance, is essential to the long-term development of small molecule inhibitors.

Oxidative stress is generated by normal cellular metabolism or produced via exogenous chemicals (such as hydrogen peroxide) and can induce several types of DNA damage including DNA base damage and the formation of apurinic/apyrimidinic (AP) sites, which are thought to be processed primarily through the base excision repair (BER) pathway (Eide et al., 1996; Swanson et al., 1999). In the BER pathway, a damaged base is removed by a specific N-glycosylase (Ntg1 and Ntg2), (Alseth et al., 1999; Meadows et al., 2003; Griffiths et al., 2009), and the resulting AP site is cleaved by an AP endonuclease. Following the processing of the 5′ terminus by deoxyribose phosphodiesterase, DNA polymerase fills in the gap and DNA ligase seals the ends together (Supplementary Figure 1). The other main DNA repair pathway is the nucleotide excision repair (NER) pathway, which generally removes bulky DNA lesions and is also implicated in the repair of oxidative stress damage (Huang et al., 1994; Boiteux and Jinks-Robertson, 2013). The damaged DNA bases are removed by introducing nicks 5′ and 3′ to the DNA damage. The 3′ incision is produced by *RAD2* and the 5′ incision by the *RAD1-RAD10* complex (Habraken et al., 1993). Once the oligonucleotide including the damaged DNA is removed, DNA polymerase fills in the gap and DNA ligase joins the ends (Supplementary Figure 1). Recombination is involved in the repair of single or double stranded breaks. Cells deficient in the Rad52 epistasis group of proteins are highly sensitive to the killing effects of agents that produce strand breaks (Mortensen et al., 1996; Pâques and Haber, 1999; Symington, 2002). Recombination rates are known to increase upon exposure to hydrogen peroxide, playing a role for recombination in response to oxidative induced DNA damage. There is an overlap between the different DNA repair mechanisms and DNA damage tolerance pathways in the processing of oxidative DNA damage (Giglia-Mari et al., 2011).

In the model yeast *Saccharomyces cerevisiae*, the removal of oxidative stress induced damage is thought to be conducted primarily through the base excision pathway (Farrugia and Balzan, 2012). The main genes involved are *NTG1* and *NTG2*, N-glycosylase-associated apurinic/apyrimidinic lyases that recognize a wide variety of damaged pyrimidines and *APN1*, a major AP endonuclease in yeast, with the majority of AP sites *in vivo* processed via this pathway (Ramotar et al., 1991; Ildiko et al., 2000; Ishchenko et al., 2005). In general, the majority of yeast cells lacking these three genes are hyper-recombinogenic and exhibit a mutator phenotype but are not sensitive to all oxidative stress chemicals. However, the additional disruption of *RAD52* confers a high degree of sensitivity to oxidative stress damage by eliminating the nucleotide excision repair pathway.

In this study, we transformed *S. cerevisiae* cells, which are exquisitely sensitive to oxidative stress, with a *C. glabrata* genomic library and isolated oxidative stress resistant clones and identified the genes that encoded mediators of oxidative stress resistance. We have identified a novel *C. glabrata* protein, CAGL0G06710g, designated *ORI1* (Oxidative stress Resistance Increased) for this work, (Table 1) which can confer oxidative stress resistance in *S. cerevisiae*. *ORI1* is a *C. glabrata* specific protein, which has no homology to proteins encoded in any other sequenced genome. We hypothesized that this gene plays a role in oxidative stress resistance in *C. glabrata* via comet assays and genetically interacts with genes playing a role in the base excision via SGA screens in the presence and absence of oxidative stress (H_2_O_2_) and nucleotide excision DNA damage repair pathways.

**TABLE 1:** *Candida* glabrata genes identified in ORFeome screen.

***C. glabrata* ORF**	***S. cerevisiae* ORF**	**Identity**	**Number of hits**
**CAGL0G06710g**	**N/A**	**99%**	**34**
**CAGL0E06094g**	**N/A**	**99%**	**9**
**CAGL0H04059g**	**N/A**	**99%**	**2**
**CAGL0A00649g**	**N/A**	**100%**	**2**
CAGL0H05181g	VPS28	99%	7
CAGL0E00583g	TRX3	93%	2
CAGL0G03531g	SPR6	99%	8
CAGL0G01848g	SNT309	100%	2
CAGL0J11220g	RPS3	98%	2
CAGL0F02673g	RPB7	98%	3
CAGL0J09482g	RMI1	98%	3
CAGL0L09042g	HAT1	68%	2
CAGL0C05137g	GPD2	75%	2
CAGL0K01683g	GPD1	73%	4
CAGL0D03630g	CET1	99%	2
CAGL0I07975g	BRX1	82%	8

*Following the transformation of the *C. glabrata* ORFeome into *S. cerevisiae*, a number of colonies were identified as being resistant to oxidative stress when screened on hydrogen peroxide. The plasmids were recovered and sequenced to identify the ORFs conferring increased levels of oxidative stress resistance. 16 ORFs were identified, 4 of which are unique to *C. glabrata* (highlighted in bold).*

## Materials and Methods

### Screening of *Candida glabrata* ORFeome

The *C. glabrata* ORFeome (Ho and Huvet, unpublished), was generated using the Gateway cloning system, whereby pENTRY plasmids were generated for each ORF in the *C. glabrata* genome and shuttled to destination vectors of interest using the LR clonase protocol, for this work the destination plasmid used was pAG423GPD-ccdB, a constitutive promoter with HIS3 selection (Ho and Haynes, 2015). The *C. glabrata* ORFeome was pooled and transformed *en mass* into *S. cerevisiae* strain BY4741, using the lithium acetate method (Gietz and Schiestl, 2007). Transformants were recovered from selective media plates containing 5 mM hydrogen peroxide, a concentration known to inhibit growth in *S. cerevisiae*. The individual colonies were isolated and grown on fresh media containing 5 mM hydrogen peroxide to remove any break through colonies. Using the QIAprep Spin miniprep kit (Qiagen), the plasmid DNA was recovered and sent for sequencing to identify the *C. glabrata* ORFs conferring stress tolerance.

### Generation of *Candida glabrata* Deletion and Over Expression Strains

The *KanMX* cassette was amplified from plasmid pFA6-kanMX4 with complementary up and down stream sequences for *ORI1*, (Supplementary Table 1 for primers used), using the following conditions for a 50 μl reaction; 1× Taq buffer, 0.2 μM dNTPs, 0.5 μM of each primer, 1 μl Taq-polymerase and plasmid DNA; 93°C for 3 min, with 30 cycles of 93°C for 30 s, 50°C for 30 s, 72°C for 2 min with 10 min at 72°C. The fragments were gel-purified in 0.7% agarose gels and the final deletion construct purified via ethanol precipitation.

### Transformations

*Candida glabrata* CAGL0G06710g was cloned into the pDONR221 entry vector using GATEWAY cloning techniques and were shuttled into pAG423GPD-ccdB destination vector (Addgene) using the LR clonase reaction. Destination vectors were transformed into *C. glabrata* via electroporation (Istel et al., 2015) and *S. cerevisiae* cells via the LiAc transformation method (Gietz and Schiestl, 2007). The correct transformants were selected for growth on SC-HIS media. Three independent transformants of each strain were collected.

### Comet Assays

Aliquots of 10^6^cells/ml *C. glabrata* and *S. cerevisiae* cells were collected by centrifugation and mixed with 1.5% low melting point agarose (w/v) in S buffer containing 2 mg/ml zymolyase (20T), 80 μl of this mixture was spread over a slide coated in 0.5% normal melting point agarose (w/v) and covered with a coverslip and incubated for 20 min at 30°C for cell wall enzymatic degradation, following which the cover slips were removed. All remaining steps were performed at 4°C. The slides were incubated in lysis solution (30 mM NaOK, 1M NaCl, 0.05% lauryl sarcosine (w/v), 50 mM EDTA, 10 mM Tris–HCl, pH10) for 20 min in order to lyse spheroplasts. The slides were then washed three times for 20 min each in electrophoresis buffer (30 mM NaOH, 10 mM Tris–HCl, pH10) to remove the lysis solution. The slides were then electrophoresis in the same buffer for 10 min at 0.7V/cm. After electrophoresis, the slides were incubated in neutralization buffer (10 mM Tris–HCl, pH7.4) for 10 min, followed by 10 min in 76 and 96% ethanol, respectively. The slides were then stained with ethidium bromide (10 μg/ml) and visualized using a Leica microsystem DM fluorescence microscope. Each condition was repeated in triplicate with 20 representative images of each slide acquired. The images were analyzed using Comet Score software.

### SGA Screens

Synthetic Genetic Array (SGA) screens were performed in triplicate with double spotting within each triplicate. The *S. cerevisiae* deletion mutant library was arrayed using a Singer RoToR HDA (Singer Instruments). For the genome-wide synthetic lethal SGA screens (SL-SGA) the MATα query strain Y7092^1^ was transformed with the *C. glabrata* gene of interest, *ORI1*. The resulting query strain was mated with the *S. cerevisiae* MATa deletion mutant array library and SGA methodology was used (Hin et al., 2001; Measday et al., 2005). For the screens in the presence of stress, the last plate of the screen (double mutant plates) was then spotted onto media containing oxidative stress (2 mM H_2_O_2_) and incubated for 24 h at 30°C. To identify deletion mutants that showed growth defects due to the expression of the *C. glabrata* genes, all screens were performed in triplicate. Growth was scored for three main criteria, slow growth (SS), lethality (SL or suppression S, improved growth) after 24 h on the final DMA plates. For verification, any potential genetic interactions had to be identified in a minimum of two out of the three replicated in any screen.

### Dotty Plates

Dot assays were performed by spotting 5 μl of 10-fold serial dilutions (OD600 = 0.1, 0.01, 0.001, 0.0001) onto specified media, and sealed plates were incubated at 30°C for 24 h. All dot assay experiments were repeated in triplicate using three different isolates of each strain.

## Results

### Identification of *C. glabrata* ORFs Mediating Oxidative Stress Resistance by Genetic Complementation

*Saccharomyces cerevisiae* is exquisitely sensitive to oxidative stress. We hypothesized that expression of effectors of *C. glabrata* stress resistance in *S. cerevisiae* would lead to increased oxidative stress resistance. We transformed, *en-bloc*, our recently constructed *C. glabrata* partial ORFeome (∼3,000 genes) into *S. cerevisiae* strain BY4741 and screened the transformants on media containing the oxidative stress agent, hydrogen peroxide (5 mM). From this we identified 1,500 stress resistant colonies, which were then individually regrown on hydrogen peroxide containing media to remove break-out colonies. The plasmids were recovered from each of these and their *C. glabrata* ORFS identified by sequencing. A total of 16 *C. glabrata* ORFs were isolated (Table 1). Recovery of genes encoding known stress protectant proteins e.g., *GPD1, GPD2*, and *TRX3* (Albertyn et al., 1994; Trotter and Grant, 2005; Tkach et al., 2012) were predicted and validated the integrity of the screen. In addition, we identified a number of genes encoding proteins with general roles in transcription/translation, epigenetic modification and endosomal sorting e.g., *BRX1, CET1, HAT1, RPB7*, *SNT309*, and *VPS28* whose over-expression are likely to have pleiotropic effects and hence deciphering a specific role in stress resistance would be extremely difficult.

Finally, we isolated four *C. glabrata* specific ORFs, encoding proteins of unknown function, *CAGL0G06710g, CAGL0E06094g, CAGL0H04059g*, and *CAGL0A00649g* (Table 1). We have taken ORF *CAGL0G6710g* forward for further investigation and for the purpose of this work refer to it as *ORI1* (Oxidative Stress Resistance Increased). It was isolated in 34/85 of the stress resistant mutants.

### Confirmation of the Role of *ORI*1 in Stress Resistance

**FIGURE 1:**
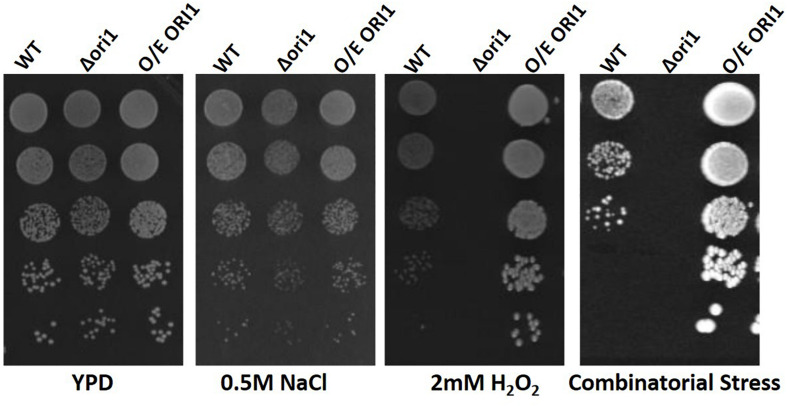
Spot plate assay of *ORI1* on stress media. *C. glabrata* wild-type, *Ori1* (CAGL0G06710g) deletion and overexpression strains. Strains were serially diluted and 5 μl spots were placed onto minimal media containing either oxidative stress (2 mM H_2_O_2_), Osmotic stress (0.5 M NaCl) and combinatorial stress (2 mM H_2_O_2_ and 0.5 M NaCl). The plates were then incubated for 24 h at 37°C. Each spot assay was performed in triplicate.

To confirm that the expression of *ORI1* conferred oxidative stress protection in *C. glabrata*, following its identification in the *S. cerevisiae* screen, we generated an *ORI1* deletion strain and over-expression strain in *C. glabrata* wild-type strain CBS138. This also confirmed that the gene is not essential for viability. The strains were then screened on oxidative (2 mM hydrogen peroxide), osmotic (1.5 M sodium chloride) and combinatorial stress plates [oxidative (H_2_O_2_) and osmotic (NaCl)] (Figure 1). The *C. glabrata*Δ*ori1* mutants are sensitive to combinatorial stress, whereas the *ORI1* over-expressing strains had an increased combinatorial stress tolerance but most striking was the increased tolerance to oxidative stress. This demonstrated a potential role for *ORI1* in mediating *C. glabrata* stress resistance.

**FIGURE 2:**
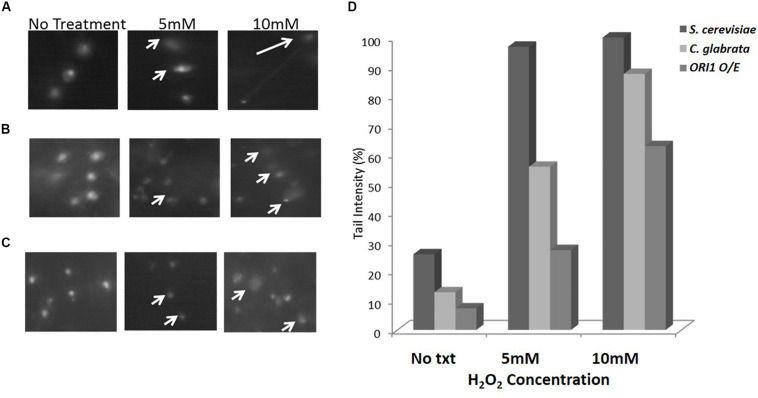
Comet assay on *C. glabrata* and *S. cerevisiae* wild-type and *ORI1* overexpressing strains. Comet assays were performed on **(A)** *S. cerevisiae* wild-type cells, **(B)** *C. glabrata* wild-type cells, and **(C)** *S. cerevisiae* cells overexpressing *ORI1* in the presence of no stress (No Txt in figure), 5 mM H_2_O_2_ and 10 mM H_2_O_2_. DNA damage was measured in 100 comets for each strain time for each condition and analysis with Comet Assay V4.0. **(D)** The DNA damage is represented as a mean ratio of tail length to head intensity allowing for positional variations in intensity. 100 comets were scored per experiment for each concentration.

Following from the spot assay, we utilized Comet assays as an uncomplicated and sensitive method to detect DNA damage at the level of individual cells. The term “comet” refers to the pattern of DNA migration through the electrophoresis gel after exposure to a DNA damaging agent, in this study, hydrogen peroxide. The intensity of the comet tail relative to the head reflects the number of DNA strand breaks. The basis for this is that loops containing a break lose their supercoiling and become free to extend toward the anode during electrophoresis. We examined *C. glabrata* and *S. cerevisiae* wild-type strains and ones over-expressing *ORI1* (Figure 2). As can be clearly seen the over-expression on *ORI1* offers protection from the DNA damage induced by the oxidative stressor H_2_O_2_ at a range of different concentrations when compared to the level of damage experienced by the wild-type cells due to the shortened tail lengths observed. This effect was most pronounced in the *S. cerevisiae* cells overexpressing *ORI1*. This may be due to the fact that *S. cerevisiae* is sensitive to oxidative stress. Control cells (i.e., no exposure to hydrogen peroxide) were also observed to display comet-like features, this is likely due to initial DNA damage present, replication forks or damage induced by the handling of cells during preparation of comets. The use of this methodology is interesting, as the combination of yeast cells, either pathogenic or non-pathogenic and the comet assay is an extremely accessible technique for genotoxicity testing.

### Synthetic Genetic Interaction Maps for ORI1 Differ in the Presence of Oxidative Stress

Synthetic Genetic Array screens enable the construction of double mutants on a genome wide scale and as such this trend for large scale genetic screens reveal a wealth of genetic interaction information on biological systems. Therefore we performed unbiased SGA screens on *ORI1* in the *S. cerevisiae* background in order to determine what genes it genetically interacts in both the presence and absence of oxidative stress (2 mM hydrogen peroxide). Interactions were identified by slow growth (synthetic sick) or death [synthetic lethality (SL)]. These genetic interactions arise from mechanistically distinct genetic origins, as the interactions are occurring between members of the same pathway. Using the principle that an increased amount of an exogenously expressed substrate may cause a fitness defect in a mutant background, this methodology has proven successful in identification of downstream targets as well as targets of specific proteolytic pathways (Costanzo et al., 2010). Therefore using this methodology can identify genetic interactions previously unknown especially when exogenous genes from yeast with no high throughput screening systems can be expressed in a model system such as *S. cerevisiae.* A SL or synthetic sick genetic interaction is defined as the combination of two genes, each individually viable, that cause a fitness defect not predicted from the multiplicative effect of combining the single mutations (either deletions or over-expression). Classically, this type of genetic interaction is that the cell cannot tolerate the disruption of a shared function of the two genes, implying that the encoded proteins work in parallel to one another. Conversely, SL genetic interaction profiles can predict genes that function in the same pathway or protein complex.

**FIGURE 3:**
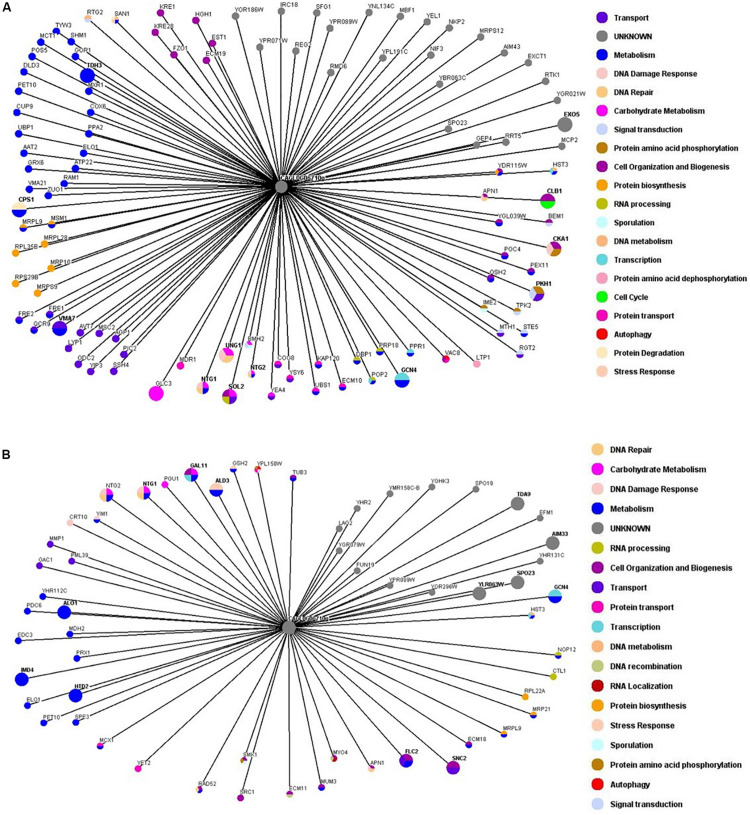
Genetic interaction network maps of *ORI1* in the presence or absence of oxidative stress. Genome wide synthetic interaction SGA screens were performed using the query strain that was transformed with *ORI1* (CAGL0G06710g) from *C. glabrata*. Genes are represented by nodes that are color-codes according to the *S. cerevisiae* database (SGD) cellular roles (www.yeastgenome.org). The interactions are represented by edges. All screens were performed in triplicate with double spotting on each plate. Deletion mutants that displayed a synthetic sick or synthetic lethal interaction are detailed in Supplementary Table 1. **(A)** Genetic interaction map of *ORI1* in the absence of stress. **(B)** Genetic interaction map of *ORI1* in the presence of oxidative stress (2 mM H_2_O_2_). It can be seen that the presence of oxidative stress affects the genes that *ORI1* genetically interacts with.

In an attempt to identify the genetic interactors of *ORI1* in *C. glabrata*, we performed genome-wide SGA screens. In brief the *S. cerevisiae* query strain containing *C. glabrata ORI1* was mated to the yeast deletion mutant arrays and the SDL methodology was used to incorporate the plasmid into the deletion mutants. Growth of the deletion mutants containing the plasmids was scored for sickness or lethality. We identified 14 synthetic lethal interactions (Supplementary Table 2) including *alo1* (*ALO1* is a mitochondrial membrane protein that catalyses the last reaction in biosynthesis of the antioxidant D-ascorbate). This allowed us to hypothesize that *ORI1* may play a role in protection from oxidative damage. In addition to *ALO1*, there were also genetic interactions with genes that play a specific role in the DNA damage and repair pathways namely *CRT10, NTG1, RAD52*, and *ECM11* (Supplementary Table 2), these genes identified in both the screens in the absence and presence of oxidative stress. The SGA screens also highlight the different genetic interactors involved in the presence of oxidative stress (Figure 3 and Supplementary Table 3). The synthetic lethal interactions (Supplementary Table 3) remained the same under both conditions indicating that the cells may be primed to encounter a stress.

### Interaction With DNA Damage Repair Pathways

**FIGURE 4:**
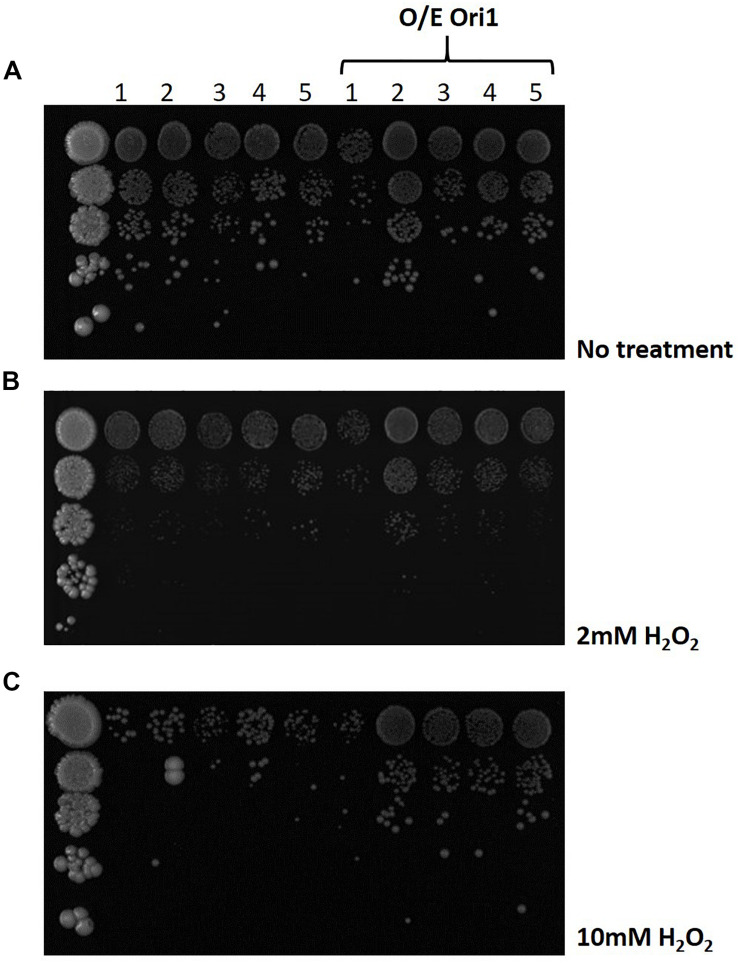
Screening of DNA pathway deletion mutants overexpressing *ORI1* on oxidative stress media. A series of *S. cerevisiae* knock-out strains for different elements of the DNA damage repair pathways were screened on media with **(A)** no stress, **(B)** 2 mM H_2_O_2_ and **(C)** 10 mM H_2_O_2_. The different deletion mutants were 1: Δntg1 ntg2 apn1 (BER pathway); 2: Δ*ntg1 ntg2 apn1 rad1* (NER pathway), 3: Δ*ntg1 ntg2 apn1 rad52* (Recombination pathway); 4: Δ*ntg1 ntg2 rad1* and 5: Δ*ntg1 ntg2 apn1 rev3* (BER pathway). Plates were spotted with 10 fold serial dilutions of cells and incubated at 30°C for 24 h. Wild-type cells were used as a control.

Following the identification of the genetic interactions with genes that play a key role in the BER and NER pathways (Supplementary Figure 1), we obtained the deletion strains from the lab of Prof Jinks-Robertson (Swanson et al., 1999) in the *S. cerevisiae* background, where the main elements of each pathway were deleted. Using these backgrounds we were able to transform *ORI1* to determine if the overexpression of this gene can compensate for the removal of these pathways (Figure 4A). As can be observed in media with no treatment (i.e., no hydrogen peroxide added), many of the mutants already exhibit a growth defect, due to the loss of different components of the DNA repair pathways which in turn was exacerbated upon exposure to hydrogen peroxide (Figures 4B,C). However, upon the addition of *ORI1*, into the different deletion strains we can observe some recovery of the sickness phenotype notably in the quadruple deletion strain, Δ*ntg1*Δ*ntg2*Δ*apn1*Δ*rad1*, on minimal media and media with increasing amounts of hydrogen peroxide as an agent of inducing oxidative stress damage. There is no improvement in the fitness of triple knockout, Δ*ntg1*Δ*ntg2*Δ*apn1*, upon the introduction of *ORI1* indicating that the exogenous gene may be hindering the other DNA damage pathways compensating for the partial lack of *ntg1, ntg2 and apn1*. The mutant background with the most significant improvement upon exposure to hydrogen peroxide when transformed with *ORI1*, was the quadruplicate knockout, Δ*ntg1*Δ*ntg2*Δ*apn1*Δ*rad1*, which is generally highly sensitive to oxidative stress and has had the nucleotide excision repair pathway deleted.

## Discussion

The natural environments for *C. glabrata* and *S. cerevisiae* are significantly different. *C. glabrata* is a commensal of mucosal surfaces where it encounters phagocytic cells from the immune system and competition from other microbes for nutrients. These environments have selected for evolution of its high resistance to numerous stress conditions such as drug exposure, temperature shifts, osmotic and oxidative stresses. *C. glabrata* has evolved the capabilities to cope with oxidative stress and can proliferate inside phagocytes when engulfed, currently the mechanism to suppress reactive oxygen species production by phagocytes is unknown. The strong resistance to oxidative stress in *C. glabrata* is believed to be mediated through the functions of a catalase (CgCta1), two superoxide dismutases (CgSod1 and CgSod2) as well as the glutathione and thioredoxin detoxification systems (Cuéllar-Cruz et al., 2008). Our current knowledge of the oxidative stress response (OSR) in *C. glabrata* has increased significantly in the last decade with the characterization of the core elements as mentioned above in addition to key transcription factors Yap1, Skn7 and the general stress response regulators Msn2 and Msn4 and the newly identified Ada2 (Yu et al., 2018). However, this current understanding of the OSR in *C. glabrata* is far from comprehensive with additional genetic components and molecular mechanisms that remain to be explored.

Knowing that *S. cerevisiae* is intrinsically sensitive to oxidative stress (Farrugia and Balzan, 2012), we utilized this trait as a model to identify oxidative stress resistant genes in *C. glabrata.* Due to their relative closeness, the need for codon optimization was nullified, allowing for the transformation of the *C. glabrata* ORFeome *en masse* into the *S. cerevisiae* background. The identification of the four novel *C. glabrata* isolates that have no sequence or synteny homology to any *S. cerevisiae* genes, highlighted potential ORFs that are conferring these increased levels of oxidative stress resistance to this human fungal pathogen. We confirmed the phenotype of one *C. glabrata* specific ORF on *CAGL0G06710g (ORI1)* as it resulted in the strongest phenotype on increasing levels of oxidative stress exposure. Following the comet assays which showed that the expression of *ORI1* in *S. cerevisiae* were offering a level of protection to DNA damage, this lead us to hypothesize that these *C. glabrata* specific genes may also play a role in DNA damage/repair pathways. DNA damage repair pathways in yeast have been extensively studied (Boiteux and Jinks-Robertson, 2013), however, this does not explain the resistance to DNA damaging agents from oxidative stress in the human fungal pathogen *C. glabrata*. Our unbiased *ORFeome* screen, which resulted in the identification of *C. glabrata* specific genes may add to this fungal pathogens story. These genes are unique to the genome of *C. glabrata*, potentially having evolved as the fungi carved out its niche as a pathogen.

Utilizing the SGA methodology to systematically screen the yeast deletion mutant array we sought to identify genetic interactions of *ORI1*. While phenotypic analysis of mutants can provide an important tool to define gene function, the effect of gene under- and over-expression when assessed globally demonstrates high levels of genetic redundancy in the yeast genome. This redundancy has resulted in the powerful approach of SGA screens to study gene function by identifying genetic relationships between genes. This is based on the knowledge that combinations of endogenous and exogenous genes that reduce cell fitness can pinpoint a shared function. With the aid of SGA screening we were able to show that *ORI1* genetically interacts with the main genes involved in DNA repair (*NTG1, NTG2 and APN1; p*-value 7.99E-05, see Supplementary Table 3) in both the presence and absence of oxidative stress. In the presence of stress, (i.e., oxidative stress), we observed an overrepresentation of genes playing a role in base-excision repair (*p*-value 0.0002993), oxidation-reduction processing (*p*-value 0.001902).

To gain insight into the role of *ORI1* in DNA damage repair in response to oxidative stress exposure, we obtained a series of deletion mutants with specific pathways deleted in the *S. cerevisiae* background (Supplementary Figure 1 and Figure 4). The removal of oxidative stress damage from yeast DNA is believed to be primarily through the base excision repair pathway. In general, cells lacking Ntg1, Ntg2, and Apn1 are not shown to be sensitive to hydrogen peroxide but upon the additional disruption of *RAD52* there is an increased sensitivity to such oxidative stress elements. These mutants were previously determined to have a growth defects, therefore through the addition of *ORI1*, we were able to circumvent this defect in the presence of oxidative stress. We observed that the quadruplicate deletion strain Δ*ntg1*Δ*ntg2*Δ*apn1*Δ*rad1* had increased resistance to increasing hydrogen peroxide upon the addition of *ORI1*. This quadruplicate deletion strain is defective in the nucleotide excision repair pathway and base excision repair pathway, and is therefore sensitive to hydrogen peroxide. With both of these pathways defective, the DNA damage tolerance pathways (recombination and translesion synthesis) must deal with exposure to oxidative stress and the resulting DNA damage. This also suggests that BER and NER are competing pathways in *C. glabrata.*

Currently, we are GFP-tagging *ORI1* to determine where it localizes to in the cell in the presence and absence of oxidative stress and if this can be correlated to its genetic interactions with *NTG1, NTG2, APN1* and *RAD52.* In addition, with the GFP-tagged strains we are building the protein interaction networks in the presence of oxidative stress in both *C. glabrata* and *S. cerevisiae.* Notwithstanding, the specific role Ori1 plays in oxidative stress resistance, it is essential for this resistance and therefore has potential as a therapeutic target being further investigated.

## Data Availability Statement

All datasets generated for this study are included in the article/Supplementary Material.

## Author Contributions

H-lH and YC performed the initial screen to identify *C. glabrata* isolates. YC and JU performed comet assays. JU, YC, and VA performed SGA screens. JU, H-lH, and KH initial concept. JU, YC, VA, and H-lH data analysis. JU wrote the manuscript. All authors contributed to the article and approved the submitted version.

## Conflict of Interest

The authors declare that the research was conducted in the absence of any commercial or financial relationships that could be construed as a potential conflict of interest.

## Footnotes

1 http://sites.utoronto.ca/boonelab/sga_technology/

## Supplementary Material

Supplementary Figure 1 Schematic of DNA repair pathways. The “X” represents DNA damage as either a base that is recognize and removed by with the base excision repair pathway (BER) or the nucleotide excision repair pathway (NER). In recombination pathway the polymerase is blocked thus blocking replication and allowing for recombination to occur. (Modified from Swanson et al., 1999).

Supplementary Table 1 Primers used in this work for the generation of *ORI1* deletion strains and ORF amplification for plasmid construction.

Supplementary Table 2 Analysis of SGA screen with *ORI11* (CAGL0G06710g) as the gene of interest.

Supplementary Table 3 Analysis of the SGA screenings with *ORI1* (CAGL0G06710g) in the presence and absence of oxidative stress in the form of hydrogen peroxide.
