# Supplementary figures and images for: Functional Characterization of a Novel Oxidative Stress Protection Protein in the Pathogenic Yeast Candida glabrata
Source: Front Genet. 2020 Sep 25;11:530915. doi: 10.3389/fgene.2020.530915 (PMC7545072; doi:10.3389/fgene.2020.530915)

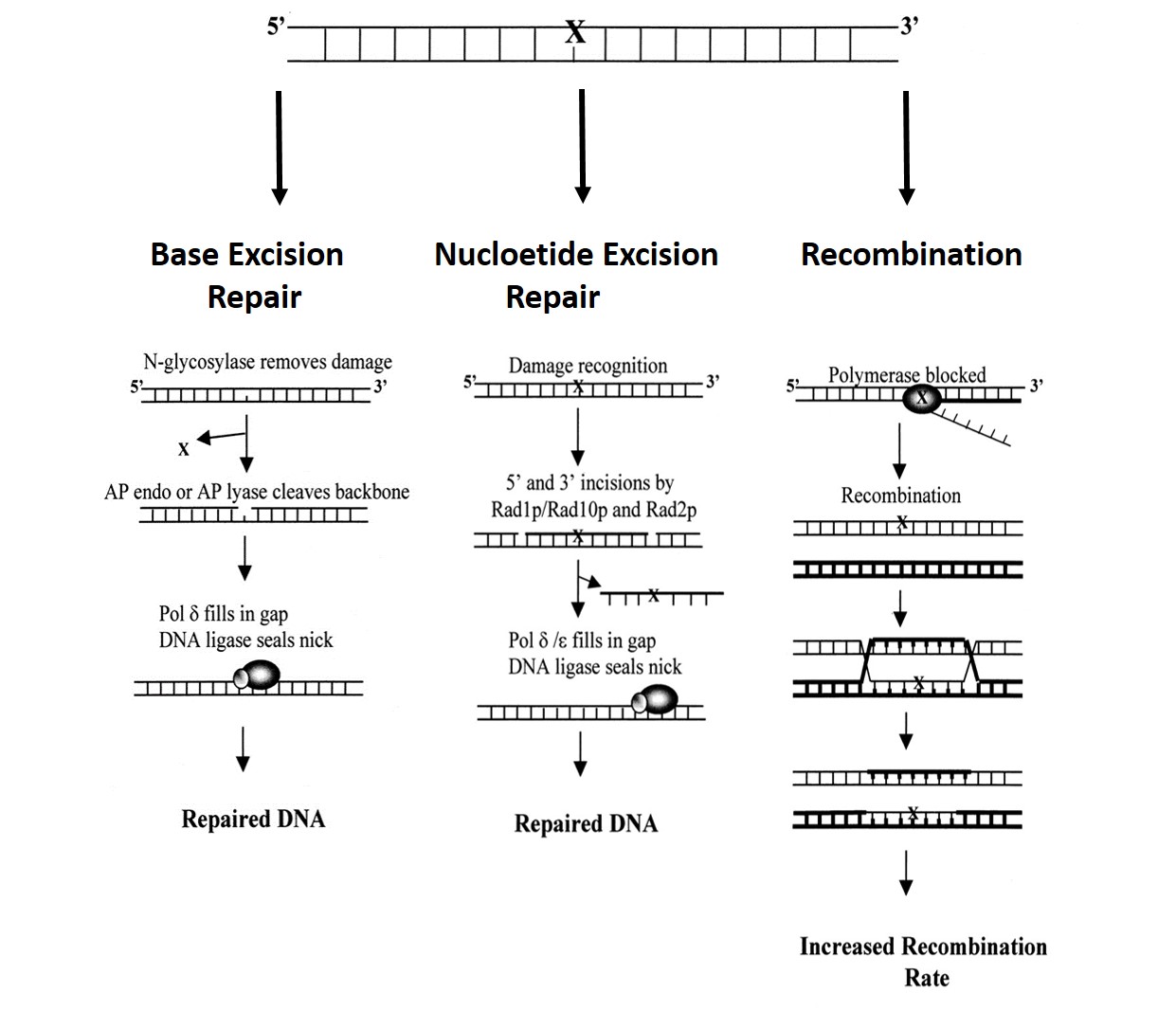

Supplement: Supplementary Figure 1 — Schematic of DNA repair pathways. The “X” represents DNA damage as either a base that is recognize and removed by with the base excision repair pathway (BER) or the nucleotide excision repair pathway (NER). In recombination pathway the polymerase is blocked thus blocking replication and allowing for recombination to occur. (Modified from Swanson et al., 1999). [file Image_1.JPEG]
